# Multi-Person Brain-To-Brain Interfaces: Ethical Issues

**DOI:** 10.3389/fnins.2019.01177

**Published:** 2019-11-05

**Authors:** Elisabeth Hildt

**Affiliations:** Center for the Study of Ethics in the Professions, Illinois Institute of Technology, Chicago, IL, United States

**Keywords:** brain-to-brain interface (B2B), brain-computer interface, ethics, autonomy, brain communication, informed consent

While the idea of a network of brains directly communicating via brain-to-brain interfaces (BBIs) may sound like science fiction to some, it actually is not. BBIs allow for technology mediated direct communication between two brains without involving the peripheral nervous system. They consist of two components: a brain-computer interface (BCI) that detects neural signals from one brain and translates them to computer commands, and a computer-brain interface (CBI) that delivers computer commands to another brain.

In a recent publication, Jiang et al. (2019) presented the first multi-person non-invasive direct BBI in which three persons used an interface called BrainNet to solve a task resembling a Tetris game collaboratively. Two participants were considered “senders,” their brain signals were recorded using electroencephalography (EEG) and, after a decoding and translation process, sent to the third person in the network, the “receiver.” The senders' decisions on whether or not to rotate a block in the Tetris-like game was submitted via pulses of transcranial magnetic stimulation (TMS) to the receiver's occipital cortex. In the case of a “yes” response, the receiver perceived a flash of light, i.e., a phosphene. Based on the phosphene experience, the receiver decided whether or not to turn the block, using an EEG interface. The experimental task also included a feedback loop through which the senders could give feedback on whether they agreed with the receiver's decision. By varying the senders' information reliability, the experiment also showed that the receiver was able to learn which of the senders was more reliable, based solely on brain-to-brain communication.

The study by Jiang et al. presents proof of concept that collaborative problem solving using multi-person BBIs is possible. The study is related to other recent research on direct BBIs (Grau et al., 2014; Rao et al., 2014; Lee et al., 2017), in particular to research in which two or three non-human primates where able to engage in common motor behavior (Ramakrishnan et al., 2015).

The approach described can encompass more than three individuals and thus provides the basis for direct brain-to-brain communication involving networks. The authors state (Jiang et al., 2019, p. 1): “Our results raise the possibility of future brain-to-brain interfaces that enable cooperative problem solving by humans using a ‘social network' of connected brains.” When talking about connected brains, the authors allude to social networks and similarities between multi-person BCIs and social networks.

It is important to stress that the study involved eliciting phosphenes in the receiver which prompted binary yes-no-responses, there was no “mind reading” or more complex information transfer. Jiang et al. (2019) mention that they are exploring the use of functional magnetic resonance imaging (fMRI) as an avenue to overcome this limitation in information complexity which would increase the bandwidth of brain-to-brain communication. Furthermore, they also consider the use of TMS to stimulate the receiver's higher-order cortical areas worth exploring in order “to deliver more complex information such as semantic concepts” (Jiang et al., 2019, p. 8). Transcranial focused ultrasound (tFUS) is another brain stimulation modality that has been used to transmit information in BBIs (Lee et al., 2017).

While the authors of the study do not reflect on the opportunities and risks of possible future uses of multi-person non-invasive direct BBIs, it is essential to widen the perspective beyond purely technical aspects to also consider possible future applications of this research and the ethical and social implications (Specker Sullivan and Illes, 2018). Even though the current state of BBI technology is far from allowing complex brain-to-brain communication, it is reasonable to take ethical aspects into consideration early on, as this supports the development of technology beneficial to individuals and society.

## Central Concepts

What are possible future contexts of use for multi-person non-invasive direct BBIs? In the medical field, they could serve as assistive devices for paralyzed patients or persons with locked-in syndrome that allow direct brain communication to exchange messages with others. As suggested by Jiang et al. (2019), a large network of individuals connected via BBIs and a cloud-based server is conceivable. This could result in a social network, similar to current social media networks. In addition, possible future applications include gaming, enhancement, user state monitoring, or encryption and silent commands, for example in military contexts (see Van Erp et al., 2012; Cinel et al., 2019; Steinert and Friedrich, 2019). It is imaginable that the technology could be useful in situations in which the collaboration of a number of specified individuals is required and in which the requested result can only be achieved when everybody contributes correctly.

The ethical issues arising in multi-person BBIs considerably overlap with those in two-person BBIs and BCIs, and involve aspects related to safety, agency, shared control, accountability, privacy, identity, self-concept, and extended mind (Fenton and Alpert, 2008; Trimper et al., 2014; Hildt, 2015; Burwell et al., 2017; Pais-Vieira and Pais-Vieira, 2018; Cinel et al., 2019; Steinert and Friedrich, 2019). While being similar from a conceptual point of view, the ethical issues involved will likely be more complicated in multi-person BBIs: In networks of directly connected brains, especially in multi-person BBIs consisting of large groups of individuals, each participant may be expected to have the role of both a sender and a recipient. This complex role distribution will result in information flows that are difficult to understand. The possibility to negotiate joint understanding and joint commitment will be necessary (cf. Dingemanse, 2017), as well as an avenue to deal with joint agency. Interestingly, the Jiang et al. study already provides a feedback option from sender to receiver that allows for a rudimentary form of collective decision-making. With large networks, difficult questions relating to the influence of individual network participants and individual and collective responsibility will arise. Also, complex implications on the users' concept of self and sense of identity may be expected, as discussed elsewhere (see Hildt, 2015). Furthermore, BBIs and multi-person BBI networks raise a number of autonomy related issues. These will be discussed in the following.

## Individual Autonomy in Brain Networks?

In BBIs, autonomy can be compromised in various ways. Primarily, through the risk that information retrieved from individual brains is distributed widely among the network without the individual's agreement. It will be crucial that all participants in BBI networks participate voluntarily and give their free and informed consent. A precondition for informed consent is that all individuals—both senders and recipients—are aware of what type of signals are recorded, collected, transferred, and received, and what the implications may be. This requires informed consent forms not only to delineate the technical details but also to describe possible implications on privacy, agency, and identity. All of this may be difficult to achieve, however.

On the side of the sender, informed consent requires that senders are able to control the type and amount of brain signals to be recorded and transferred. One option may be to choose very specific brain signals and thus limit BBI functionality—as was done in the Jiang et al. study with the use of a binary signal. With more volatile BBIs such as those involving fMRI, the situation will be much more complex and it will be much more difficult to ensure the senders' autonomy. For autonomy reasons, it may be necessary to limit the volatility of BBIs and the spectrum of technologies used.

Privacy issues arise when a person's brain data is recorded and used, and the person is not aware of it or does not want the data to be recorded, transformed or distributed. This is particularly relevant when the data allows for conclusions to be drawn on the individual's condition or mental states.

Regarding the question of how to regulate neuroscience and neurotechnology, Ienca and Andorno argue in favor of a “right to brain privacy,” which “aims to protect people against illegitimate access to their brain information and to prevent the indiscriminate leakage of brain data across the infosphere” (Ienca and Andorno, 2017, p. 15). They point out that brain data retrieved from a person's brain can be considered to be “personally identifiable information” that deserves protection. With regard to privacy protection, current and also possible future advanced techniques to identify individuals have to be taken into consideration (see Rocher et al., 2019).

In order to protect individuals from unknowingly giving away sensitive information in BBIs and similar systems, there is a clear need to increase awareness of autonomy and privacy issues related to brain signals, to be transparent about what data is recorded and used and what the implications of this may be.

On the side of the receiver, individual autonomy requires that participants in multi-brain BBIs are able to control what type of information they want to receive, from whom, and when. Especially when it comes to large brain networks, a mechanism will be required to reduce noise, limit input, and suppress unwanted senders. With large networks, something similar to “like buttons” or “dislike buttons” in current social media might be imaginable, with which receivers may be able to block or reduce certain types of transmissions or senders. Interestingly, the study published by Jiang et al. already investigated this principle, i.e., the possibility for receivers to “weigh” the relevance of a sender and to rely more heavily on preferred senders.

Can receivers in BBI networks be harmed? While the signals transferred will influence their neural computation, it is unclear right now what the implications could be and whether there is a risk of overstimulation. The risks will vary considerably, depending on the technology used, the type of signal transferred, and the type of network. Also, questions related to identity have to be considered here, especially in more volatile BBIs (Hildt, 2015).

Several authors have suggested a “right to mental integrity” that allows individuals to protect their brains from potential harm (Ienca and Andorno, 2017; Lavazza, 2018). In case of potential harm, such a right could allow receivers to limit brain input in BBIs. However, on the receiver side, much more flexibility could be achieved by referring to individual autonomy and informed consent. These concepts would not be confined to protect from potential harm but would also include protection from any kind of unwanted signal.

Additional autonomy related questions arise as participants in BBI networks depend heavily on other network members and the input they provide. The role of recipients is to rely on the inputs received, to find out who are the most reliable senders, and to make decisions based on the inputs and past experiences. In this, a lot of uncertainty and guessing will be involved, especially as it will often be unclear where the input or information originally came from. For recipients in brain networks, individual or autonomous decision-making seems very difficult if not almost impossible. This is problematic in itself, not just in view of the possibility of fake news or brain hacking (Ienca and Haselager, 2016). A concept of “extended autonomy” may be conceivable here, related to the idea of extended mind and cognition (see Clark and Chalmers, 1998). Furthermore, the possibility to negotiate joint commitment, similar to what is normally done through language communication, will be crucial for collective BBI-based agency (Gilbert, 1990; Dingemanse, 2017).

## Conclusion

BBIs and BBI networks are not science-fiction any more, they are technically feasible in principle, even though at present, BBI research clearly is in its infancy. BBIs come along with a number of currently unresolved ethical issues including autonomy, privacy, agency, accountability, and identity. While it is questionable whether multi-person BBIs will have broad applications in the near future, some very specific uses seem conceivable. If research is to continue in this field, there is a clear need to start thinking right now about how to responsibly shape the development and future use of BBIs and direct brain communication. A first step in this process could be to have an interdisciplinary team of researchers develop recommendations or guidelines, possibly using a recent Portuguese document (Pais-Vieira and Pais-Vieira, 2018) as one of the starting points.

## Author Contributions

The author confirms being the sole contributor of this work and has approved it for publication.

### Conflict of Interest

The author declares that the research was conducted in the absence of any commercial or financial relationships that could be construed as a potential conflict of interest.
